# Use of preventive medication and supplements in general practice in patients in their last year of life: a Retrospective cohort study

**DOI:** 10.1186/s12875-023-02049-x

**Published:** 2023-04-15

**Authors:** Anne Antonisse, Frederieke H. van der Baan, Matthew Grant, Gon Uyttewaal, Cathelijne Verboeket, Hanneke Smits-Pelser, Saskia C. C. M. Teunissen, Eric C. T. Geijteman

**Affiliations:** 1 Centre of Expertise Palliative Care, Department General Practice, Julius Centre for Health Sciences and Primary Care, UMC Utrecht, Utrecht, the Netherlands; 2 Academic Hospice Demeter, de Bilt, the Netherlands; 3 Leidsche Rijn Julius Healthcare Centers, Utrecht, the Netherlands; 4 Department of Medical Oncology, Erasmus MC Cancer Institute, Rotterdam, the Netherlands

**Keywords:** Preventive medication use, Palliative care, Discontinuing, Life-limiting illness, Home-care setting, Inappropriate drugs

## Abstract

**Background:**

Several preventive medications and supplements become inappropriate in the last phase of life due to increased risk of adverse events caused by changed pharmacokinetics, drug-drug interactions, and changed care goals. Information on these preventive medication and supplements use in patients with a life-limiting illness in the home-care setting is limited. The primary aim of this study was to assess the use of four different groups of preventive drugs and supplements, which are inappropriate in adult patients with a life-limiting illness, living at home in the last year of life. The secondary aims were to assess reasons for discontinuing these drugs as documented in the general practitioners’ patient file and whether these reasons affected the time between medication discontinuation and death.

**Methods:**

We performed a retrospective cohort study using the routine primary care database of the Julius General Practitioners’ Network of the University Medical Centre Utrecht, a database consisting of routine care data from GPs from the city of Utrecht and its vicinity. Patients in the homecare setting with a life-limiting illness, diagnosed at least one year before death, were included. Descriptive analyses were used to describe the study population and the frequency of starting, using, and discontinuing medication and supplements in the last year of life.

**Results:**

A total of 458 of 666 included patients (69%) used at least one preventive drug in the last year of life. Vitamins were used by 36% of the patients, followed with 35% using cholesterol-lowering medication, 24% using calcium supplements and 9% using bisphosphonates. Bisphosphonates were discontinued by 70% of the users, calcium supplements by 61%, vitamins by 56% and cholesterol-lowering medication by 48% of the users, with a median interval between day of discontinuation and death of 119, 60, 110 and, 65 days, respectively. The median time between medication or supplement discontinuation and death was longest in patients with side effects and who had medication reviews.

**Conclusion:**

Many patients in their last phase of life in the home-care setting use inappropriate medication and supplements. Timely medication review may contribute to optimise medication use in the last year of life.

**Supplementary Information:**

The online version contains supplementary material available at 10.1186/s12875-023-02049-x.

## Background

Medication burden increases for patients in the last phase of life due to continuation of drugs for comorbid conditions and the addition of medications for symptom control [1]. The median number of prescriptions per patient per day is seven during the last week of life in the Netherlands, which is defined as polypharmacy (≥ 5 medications) [2, 3]. Schenker et al. evaluated the associations between polypharmacy and quality of life (QoL) in 372 patients with a life-limiting illness (LLI), demonstrating a relationship between more medications and higher symptom burdens and lower quality of life [4]. In the last phase of life some drugs can become inappropriate medications (IMs) due to 1) increased risk of adverse events caused by drug-drug interactions or changed pharmacokinetics and pharmacodynamics drug parameters [5, 6]; 2) medications’ time to benefit may exceed predicted life-expectancy [1, 5] and 3) changed care-goals [3, 5].

A questionnaire study found that 73% of physicians agreed with the statement that patients who are in the last phase of life use too many medications [7]. Despite this consensus among healthcare professionals, there seems to be a passivity towards the reduction of IMs [8].

A systematic review of qualitative research suggested that limited consultation time, fragmented care among multiple prescribers, and ambiguous of changing care goals add to the clinical complexity that prescribers are faced with [9]. Geijteman et al. also found that physicians did not consider withdrawal of certain drugs because of limited awareness, low priority, and uncertainty about the benefits and harms of continuing or discontinuing certain medications. In addition, patients may feel that healthcare professionals are ‘giving up hope’ or that they are not receiving optimal care when medication is discontinued [10, 11].

Although guidance around discontinuing medications and supplements in patients with a limited life-expectancy is scarce, there is consensus that several preventive medications and supplements, such as cholesterol-lowering medications, vitamins, calcium supplements, may be seen as inappropriate in patients last year of life [12–14].

General practitioners (GPs) frequently have long-term relationships with patients that are often intensified in the last phase of life [15]. To date, little is known about (in)appropriate prescriptions in patients in the last phase of life in the general practice setting. To our knowledge, there has been only one study conducted in the home setting determined the most utilised (preventive) medication in the last week of life of 60 patients [3].

In the Netherlands, more than one third of patients who are in need for palliative care died at home [16, 17]. Considering the significant size of this group and the scarcity of literature, it is important to assess the (in)appropriate medication and supplement use in these patients related to their aims in quality of life. The primary aim of this study was to assess the prescription, continuation and discontinuation of four preventive, and inappropriate, medication – cholesterol-lowering medication, vitamins, calcium supplements, and bisphosphonates – in adults with a LLI during the last year of life in the home-setting. The secondary aims were to assess reasons for discontinuing these drugs as documented in the general practitioners’ patient file and whether these reasons affected the time between medication discontinuation and death.

## Methods

### Study design and population

We performed a retrospective cohort study of general practitioners’ patients’ files. Data for this retrospective cohort were obtained from the Julius General Practitioners’ Network (JGPN) database, which contains routine primary care data extracted from electronic primary care settings in and around the area of Utrecht [18]. Patients were eligible for inclusion if they 1) were aged 18 years or older, 2) deceased in 2019 and 3) were diagnosed with a LLI at least one year before death. A LLI was defined as an illness which often causes a patient to receive palliative instead of curative care and will eventually lead to the patient’s death. Appendix 1 lists the diagnoses classified as life-limiting [19].

### Data collection

Data consisted of patient characteristics, clinical notes of GP consultations, coded diagnoses, and medication history over the last two years of life. Patient characteristics, which were used to describe the study population, included: date of birth, date of death, and gender. Registered diagnoses were classified according to ICPC (International Classification of Primary Care). In case of multiple coexisting LLIs, the patient was categorised according to the longest existing illness. Some of the LLI’s were misclassified in the GP’s file (e.g. when it was documented under the ICPC-code for the primary health issue rather than the definitive diagnosis), and for some diagnoses, such as frailty, there was no ICPC-code. Because of this fact, the files of patients who did not have a LLI based on ICPC-codes were screened manually for a LLI (AA, FB). Patients’ files were also screened manually for reasons for discontinuing medication around the stop date (AA). A supplement for the data collection is included in appendix 2.

### Outcomes

We defined having a LLI as having at least one ICPC-code linked to a life-limiting diagnosis registered in the patients’ file or when a LLI was established when reviewing the patients’ files manually. Our primary outcome was the use of four different groups of preventive medications and supplements in the last year of life: cholesterol-lowering medications, vitamins, calcium supplements, and bisphosphonates [13, 14]. These four medications were evaluated because these medication become inappropriate in the last year of life. Thereby, these medications are often prescribed and thus used by a large group of patients. Fibrates, even though they are cholesterol-lowering, were not included in the analyses since they are not a first-line treatment in the Netherlands and therefore hardly ever prescribed.

Corresponding Anatomical Therapeutic Chemical (ATC) classification codes are listed in appendix 3 [20]. Medication use in the last year of life was defined as having at least one prescription in the last year of life regardless of stop date. The medication that was first prescribed in the last year of life and was not a repeat prescription was referred to as ‘started with medication’. ‘Stopped with medication’ was defined as medication that had a stop date in the last 365 days before death. All drugs within one medication group had to be discontinued to be listed as ‘stopped with medication’. No distinction was made between different dosages and combinations of medications in one tablet (e.g., cholesterol-lowering simvastatin and ezetimibe).

### Data analysis

Descriptive analyses were used to describe the study population and the frequency of starting, using, and discontinuing medication in the last year of life. All analyses were conducted using IBM SPSS Statistics 26 (IBM Corporation, 2019). Patients’ files were screened manually for reasons for discontinuing medication (AA). Criteria for classifying the reasons for discontinuation is added in appendix 2.

### Ethics

This research was reviewed by the Medical Ethics Committee (METC) NedMec and not considered to the Medical Research Involving Human Subjects Act of the Netherlands (Dutch: WMO) (21–498). All participating GPs in the JPGN adequately inform their patients about the use of their medical records for research purposes through flyers and/or information on their website. Patients may opt out, and their routine care data will not be used for the JPGN database. Meaning, that patients do not opt-out for this specific study but for all studies that used the JPGN database.

## Results

### Study population

**Fig. 1:**
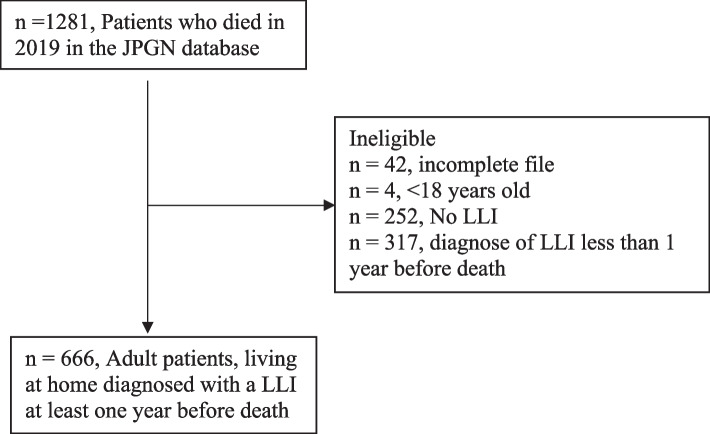
In- and exclusion flowchart

A total of 1281 patients included in the JPGN database died in 2019. Of those, 666 met the eligibility criteria for this study (Fig. 1). Table 1 displays the patient characteristics. Of the included patients, 334 (50%) were females. Age at time of death ranged from 32 to 106 years, with a median age of 82 [IQR: 74—89]. As shown in Table 1, the most frequent LLIs were cancer, followed by chronic obstructive pulmonary disease (COPD), frailty, and congestive heart failure.

**Table 1:** Patient characteristics

	**Patients with a LLI at least 1 year before death (*N* = 666)**
Sex (female), n(%)	334 (50)
Age at diagnose (year), n(%)	
< 55	20 (3.0)
55–64	47 (7.1)
65–74	116 (17.4)
75–84	216 (32.4)
≥ 85	267 (40.1)
Median age in years [IQR]	82 [74-89]
LLI, n (%)	
Cancer	227 (34.1)
Chronic Obstructive Pulmonary Disease	143 (21.5)
Congestive Heart Failure	97 (14.6)
Frailty	100 (15.0)
Dementia	61 (9.2)
Neurological^1^	22 (3.3)
Kidney failure	9 (1.4)
Liver failure	6 (0.9)
Other^2^	1 (0.2)

^1^Multiple sclerosis, ALS, Parkinson’s’ disease

^2^Congenital anomaly endocrine glands/metabolism

### Medication and supplement use

The four different groups of preventive medication and supplements that are relevant in this study consisted of a total of 32 different drugs. Table 2 shows the use of these medication subgroups over the last year of life. 458 of the 666 patients (69%) used at least one of these preventive drugs in the last year of life. Vitamins were used by 36% of the patients, followed by 35% of the patients who used cholesterol-lowering medication, 24% used calcium supplements and 9% used bisphosphonates. Cholesterol-lowering medication was stopped by 110 patients (48%) and bisphosphonates was stopped in 42 patients (70%) with a median time between discontinuation and death of 119 days. In the last year of life vitamins were started in 10% of the patients, calcium supplements by 7%, cholesterol-lowering medication by 3% and bisphosphonates by 2%, respectively.

**Table 2:** Medication and supplement use in the last year of life

Medication/supplement	Used, n (% of patient total)	Stopped, n (% of the patients that used medication)	Median time between stop and death in days [IQR]	Started in last year of life, n (% of patient total)	Median time between start and death in days [IQR]
Cholesterol-lowering medication	230 (35%)	110 (48%)	65 [23–167]	25 (4%)	200 [80 – 313]
Calcium supplements	159 (24%)	97 (61%)	60 [10–189]	48 (7%)	158 [62 – 283]
Vitamins	243 (36%)	136 (56%)	110 [40–201]	64 (10%)	173 [70 -274]
Bisphosphonates	60 (9%)	42 (70%)	119 [23 – 250]	12 (2%)	245 [153 – 328]

Patient total is 666

### Reasons for discontinuation

In Table 3 the reasons for discontinuation are summarized per group of preventive medication and supplement. The median time between discontinuation and death was longest in case of side effects (e.g., myalgia, gastrointestinal complaints) or in the context of a medication review, with 312 and 71 days, respectively. The shortest median time between discontinuation and death was when the patient was unable to take the medication (e.g., dysphagia, nausea) or when the patient was undoubtedly in the terminal stage, with a median time of 2 and 7 days, respectively.

**Table 3:** Reasons for discontinuation

	**Cholesterol-lowering medication, n (%)**	**Calcium supplements, n (%)**	**Vitamins, n (%)**	**Bisphos-phonates, n (%)**	**Median time between stop and death in days [IQR]**
Stopped in total	110 (48%)	97 (61%)	136 (56%)	42 (70%)	
Number of patients in whom the reason for discontinuing was documented	28 (25%)	23 (24%)	13 (10%)	9 (21%)	
Numer of patients in whom the reason for discontinuing was not documented	82 (75%)	74 (76%)	123 (90%)	33 (79%)	
Reasons					
*Medication review*	13 (12%)	5 (5%)	5 (4%)	2 (5%)	71 [16 -199]
*Undoubtedly in the terminal stage*	7 (6%)	13 (13%)	3 (2%)	3 (7%)	7 [3-13]
*Own initiative*	4 (4%)	4 (4%)	5 (4%)	1 (2%)	48 [15 – 122]
*Unable to take medication*	3 (3%)	1 (1%)	0	0	2 [1 – 34]
*Side effects*	1 (1%)	0	0	3 (7%)	312 [170 – 344]

## Discussion

### Medication and supplement use

This study describes preventive, inappropriate medication use during the last year of life in patients with a LLI in the home setting. It shows that of the 666 patients included ranging from 32 to 106 years of age, 458 (69%) used at least one of the preventive drugs in the last year of life. Ranging from 60 patients (9%) using bisphosphonates to 243 (36%) using vitamins.

Earlier research about medication use in 180 patients in the last week of life in patients in the homecare setting in the Netherlands found a similar percentage of vitamin users [3]. On the contrary, the use of cholesterol-lowering medication and calcium supplements differed. We found that 35% of patients used cholesterol-lowering medication in their last year of life, as opposed to Arevallo et al. findings that 3.2% of patients used it in their final week. In addition, 24% of patients used calcium supplements in their last year of life compared to 6.5% in their last week of life. A Swedish study assessed medication use in patients with cancer aged 65 years and older in the 12^th^month before death. In which 21,5% of patients using cholesterol-lowering medication, 8.2% using vitamins, 10.5% calcium supplements, and 4.2% using bisphosphonates [6].

Cholesterol-lowering medications and vitamins were discontinued by the smallest proportion of patients. Given that in literature cholesterol-lowering medication is the most widely accepted medication to discontinue in the palliative setting, it is remarkable that relatively few patients stopped using it [13, 14, 21]. Despite the clear consensus regarding cholesterol-lowering medication, there seems to be a discrepancy between literature and practice. Efforts have been made to reach similar consensus regarding other preventive medications. Delphi studies, for instance, have resulted provided some guidance for discontinuing medication in the palliative care setting [13, 14, 21].

Despite this guidance, we found that a substantial number of patients continue to use IMs in their last year of life. This may be due to limited awareness among health care providers and the clinical complexity that they are faced with when providing palliative care. The preventive drugs and supplements in this study are often appropriate in the general population, however, in the context of a palliative setting, these can become IMs [13, 14, 21]. The continuation of these medications and supplements is possibly not consistent with the goal of providing optimal palliative care and obtaining patient’s wishes in quality of life. Therefore, it is important to have a critical discussion about the use of medication with the patient and informal caregivers/loved ones, in which it can be determined whether preventive medication and supplement use is in line with the patients’ goals [22].

### Reasons for discontinuation

In the documented reasons for discontinuation, we found a difference between a reactive and a proactive approach to discontinuing medication. In a reactive approach medication are discontinued as a reaction on an issue or problem. In a proactive approach, such issues or problems may be prevented. A medication review is a proactive approach of discontinuing IM whereas the other four documented reasons (1) when the terminal stage was undoubtedly reached 2) patient’s own initiative, 3) inability to take medication and 4) side effects) have a more reactive nature. The reactive reasons for discontinuation have the shortest median time between stopping and death, except for cessation due to side effects. When the patient took the initiative to discontinue, the median time was 48 days, 7 days when the patient was undoubtedly in the terminal stage, and 2 days when the patient was unable to take medication. When medication was proactively discontinued during a medication review, it occurred earlier in the palliative phase, with a median duration between discontinuing and death of 71 days.

It was striking that proactive discontinuation during medication reviews showed a notable earlier cessation of medication and supplements in comparison to other reasons for discontinuing. This proactive intervention possibly contributes to timely discontinuation of IMs with positive consequences for quality of life. A systematic review and meta-analysis, including RCTs and non-randomized trials, compared deprescribing interventions such as medication reviews, to usual care among community-dwelling older adults. They found a significant reduction of the use of IMs when a medication review was conducted [23]. These findings underline the possible added benefit of a timely medication review in both patients with LLI as well as community-dwelling older adults.

### Strengths

This study consists of a large study population of 666 patients, which makes the results broadly generalizable. It differs on three significant points from similar studies concerning medication use in palliative care and thus contributes to the existing literature. Firstly, previous studies were often conducted in a hospital or hospice setting. Given the fact that a substantial number of the patients who are eligible for palliative care die at home, it is of added value that this study provides data on these patients [16]. Secondly, this study included all adult patients eligible for palliative care, regardless of LLI or age, while other studies focused on a specific diagnosis exclusively on older patients. Lastly, in contrast to other studies, this study assesses medication and supplement use over the entire last year of life instead of the last week(s) of life.

### Limitations

Nevertheless, our study has some limitations that need to be acknowledged. First, the retrospective nature of this study restricts us to the information that was documented. For instance, in some cases the date registered in the system as the stop date did not reflect the date on which the patient actually ceased using the medication. Thus, we cannot rule out the possibility that medication has been discontinued in practice without it being registered. The registered date of death may differ slightly form the actual date that the patient died. However, considering that this study covers a period of a year, this presumably has a limited effect. Additionally, in only a minority of patients’ files the reasons for discontinuing medication were noted. In retrospect, this might make the size of the included patient population a limitation. Second, patients admitted to a hospice or hospital for a relevant amount of time were not excluded from the analysis. Information about medication use was not available for analysis while admitted. Same goes for information about over-the-counter use of vitamins and calcium. For both, this possibly means that some medication changes were not included in the results. Third, manual review of patients’ file could have led to some bias. Nonetheless, this is expected to have a minimal effect on our findings. Lastly, due to the pseudo-anonymised nature of the database, the palliative knowledge of the prescribers is unknown.

## Conclusion

The number of patients with a LLI that use, continue and/or start with IMs during the last year of life suggests that there is room for improvement in optimising relevant medication in the palliative phase. The appropriateness of IMs are context and patient dependent. When the setting changes from curative to palliative, the appropriateness of a preventive medication and supplements asks for a shift related to quality of life as well. As such, it is of added value to provide proactive patient centred care which includes an advanced care planning process with the patient. Such conversations should include the critical consideration of whether each medication is still in line with the patients’ needs, wishes, and values. The aim of this proactive approach in a collaboration between GP, homecare nurses, patients, and caregivers is to contribute to the central goal of palliative care which is to optimise the quality of life.

## Supplementary Information

**Additional file 1: Appendix 1.** Life-limiting illnesses.** Appendix 2.** Data-collection.** Appendix 3.** ATC-codes.

## Data Availability

Unfortunately, the raw data cannot be made publicly available due to ethical and legal restrictions, as also stated in paragraph 2.2.5, privacy issues. However, all interested readers may request data from Dr. Marlous Kortekaas (m.f.kortekaas@umcutrecht.nl), the study coordinator of the JGPN.

## Abbreviations

QoL: Quality of life
LLI: Life-limiting illness
IMs: Inappropriate Medications
GP: General Practitioner
JPGN: Julius General Practitioners’ Network
ICPC: International Classification of Primary Care
ATC: Anatomical Therapeutic Chemical
COPD: Chronic Obstructive Pulmonary Disease
IQR: Interquartile range
RCTs: Randomized controlled trials
