# Supplementary material for: Use of preventive medication and supplements in general practice in patients in their last year of life: a Retrospective cohort study
Source: BMC Prim Care. 2023 Apr 15;24:101. doi: 10.1186/s12875-023-02049-x (PMC10105458; doi:10.1186/s12875-023-02049-x)
Supplement: Supplementary file 1 — Additional file 1: Appendix 1. Life-limiting illnesses. Appendix 2. Data-collection. Appendix 3. ATC-codes. [file 12875_2023_2049_MOESM1_ESM.docx]

Appendices

Use of preventive medication in general practice in patients in their last year of life: a Retrospective cohort study

Authors:

Corresponding author: Anne Antonisse, Medical student, Centre of Expertise Palliative Care, Dept General Practice, University Medical Centre Utrecht. [a.antonisse-2@umcutrecht.nl](mailto:a.antonisse-2@umcutrecht.nl)

Frederieke H. van der Baan, Epidemiologist – Centre of Expertise Palliative Care, Dept. General Practice, UMC Utrecht. [f.h.vanderBaan-2@umcutrecht.nl](mailto:f.h.vanderBaan-2@umcutrecht.nl)

Matthew Grant, GP, PhD, Palliative Medicine Physician – Centre of Expertise Palliative Care, Dept. General Practice, UMC Utrecht. [m.p.grant-2@umcutrecht.nl](mailto:m.p.grant-2@umcutrecht.nl)

Gon Uyttewaal, clinical nurse specialist palliative care and hospice care at home, Academic hospice Demeter, de Bilt. [g.uyttewaal@hospicedemeter.nl](mailto:g.uyttewaal@hospicedemeter.nl)

Cathelijne Verboeket, clinical nurse specialist palliative care and hospice care at home, Academic hospice Demeter, de Bilt. [c.verboeket@hospicedemeter.nl](mailto:c.verboeket@hospicedemeter.nl)

Hanneke Smits-Pelser, general practitioner and palliative care consultant, Leidsche Rijn Julius Healthcare Centers, Utrecht, the Netherlands. [hsmits@lrjg.nl](mailto:hsmits@lrjg.nl)
Saskia C.C.M. Teunissen, RN, PhD, professor Palliative care and hospice care, - Centre of Expertise Palliative Care, Dept. General Practice, UMC Utrecht. [s.teunissen@umcutrecht.nl](mailto:s.teunissen@umcutrecht.nl)
Eric C.T. Geijteman, Medical Oncologist, Clinical pharmacologist – Department of Medical Oncology MC Cancer Institue, Rotterdam. [e.geijteman@erasmusmc.nl](mailto:e.geijteman@erasmusmc.nl)

**Appendix 1 – Life-limiting illnesses**

Life-limiting illnesses^1^

| **ICPC-code** | **Diagnosis** |
| --- | --- |
| **Cancer** |  |
| A79 | Malignancy with unknown primary site |
| B72 | Hodgkin’s disease |
| B72.01 | Hodgkin’s disease |
| B72.02 | Non-Hodgkin lymphoma |
| B73 | Leukemia |
| B74 | Other malignancy of the blood-/lymphatic system |
| B74.01 | Multiple myeloma |
| D74 | Malignancy of the stomach |
| D75 | Malignancy of the colon/rectum |
| D76 | Malignancy of the pancreas |
| D77 | Other/unspecified malignancy of the digestive organs |
| D77.01 | Malignancy of the oesophagus |
| D77.02 | Malignancy of the salivary glands |
| D77.03 | Malignancy of the lip/mouth/tongue |
| D77.04 | Malignancy of the liver/gallbladder/bile duct |
| K72.01 | Malignancy of the cardiovascular system |
| L71.01 | Musculoskeletal malignancy |
| N74 | Nervous system malignancy |
| R84 | Bronchus/lung malignancy |
| R85 | Other respiratory malignancy |
| S77 | Skin/subcutaneous malignancy |
| S77.02 | Spinocellular / squamous cell carcinoma |
| S77.03 | Malignant melanoma |
| S77.04 | Kaposi-sarcoma |
| T71 | Thyroid malignancy |
| U75 | Kidney malignancy |
| U76 | Bladder malignancy |
| U77 | Other urinary tract malignancy |
| W72 | Malignancy related to pregnancy |
| X75 | Malignancy of the cervix uteri |
| X76 | Malignancy breast woman |
| X76.01 | Adenocarcinoma breast woman |
| X77 | Other malignancy of female genital organs |
| X77.01 | Endometrial carcinoma |
| X77.02 | Ovarian malignancy |
| Y77 | Prostate malignancy |
| Y78 | Male genital/breast malignancy |
| Y78.01 | Penile malignancy |
| Y78.02 | Testicular malignancy |
| Y78.03 | Breast malignancy |
| **Congestive Heart Failure** |  |
| K77 | Decompensatio cordis |
| **Chronic Obstructive Pulmonary Disease** |  |
| R95 | Emphysema/COPD |
| **Neurological (N)** |  |
| N86 | Multiple sclerosis |
| N87 | Parkinsonism, Parkinson’s disease |
| N87.01 | Parkinson’s disease |
| N99.01 | ALS |
| **Dementia (P)** |  |
| P70 | Senile dementia/Alzheimer’s |
| P70.01 | Alzheimer’s disease |
| P70.02 | Multi-infarct dementia |
| **Manually determined life-limiting illnesses** |  |
| **Frailty** |  |
| **Kidney Failure** |  |
| **Liver failure** |  |
| **Other** | Congenital anomaly endocrine glands/metabolism |

**Appendix 2 - Data-collection**

1. **A priori exclusion**
   1. **Age**

The registered date of death and date of deregistration did not always match. Age at time of death was determined by calculating time between date of birth and date of death or date of deregistration, whichever came first. Patients younger than 18 years of age at the time of death were excluded from the analysis.

**1.2 Life-limiting diagnoses**

The International Classification of Primary Care (ICPC) is a classification method for patient’s reason for encounter (RFE), the problem/diagnosis managed, interventions, and the ordering of these data in an episode of care structure.^2^ The dataset from the JGPN database included a table that displayed the frequencies of ICPC-codes linked to a diagnosis that were registered per patient in the last two years of life. Patients who had an ICPC-code linked to a life-limiting illness were included in the analysis. The ICPC-codes for illnesses that were considered life-limiting, are listed at the bottom of this document. If two life-limiting illnesses co-existed, we categorized the patient under whichever diagnosis existed the longest. Some of the life-limiting illnesses were misclassified and for some diagnoses, such as frailty, there is no ICPC-code. Because of this fact the files of the patients who did not have an life-limiting illness based on ICPC-codes were screened manually for a life-limiting illness. If the file indicated a life-limiting illness, patients were included. All patients who did not have a life-limiting illness based on ICPC-code or when manually reviewed, were excluded from the analysis.

**1.3 Follow-up time**

The aim of the study is to assess the medication use in patients with a life-limiting illness for the entire last year of life. Subsequently, all patients who did not have a follow-up time from diagnosis to death of at least one year were excluded. For the patients that were included based on ICPC-codes we calculated the time between first registration of the ICPC-code and the date of death. If the time between these dates was <365 days, the patient was excluded from the study. For patients who were included by manually reviewing their files, we calculated the time between first contact with the GP regarding their diagnosis and the date of death. Because first contact with the GP does not equal date of diagnosis, we used a different minimal follow-up time for these patients. Manually included patients that had a follow up time from diagnosis to death of <330 days were excluded from the analysis.

1. **Medication use analysis**For analysing the medication use in the last year of life we made a distinction between the starting, the using and the discontinuation of medication

- The medication that was first prescribed in the last year of life and was not a repeat prescription was referred to as ‘started with medication’. Medication was selected if it was the first prescription, and the start date was <365 days prior to death.
- The ‘use of medication’ was defined as all preventive medication that was used in the last year of life regardless of start- or stop date. We defined this as all preventive medication that had a stop date > date of death-365 days.
- Medication was selected as ‘stopped’ if the last prescription of the medication had a stop date <365 and >0 days prior to death. All medication within one medication group had to be discontinued to be assessed as ‘stopped with medication’.

1. **Reasons for discontinuing medications**

Patients who stopped with medication in the last year of life were identified. Per stopped medication, the GP’s notes were manually screened for reasons for discontinuation around the stop date.

Reasons for discontinuing medication were divided into five categories:

1. Discontinuation in context of a medication review
   1. The reason for discontinuing was classified in this category when a medication review was clearly stated in the patients’ file near the stop date of the medication.
2. Discontinuation because the patient requested to
   1. The reason for discontinuing was classified in this category when it was noted in the patients’ file that discontinuing the medication was the patients’ own initiative.
3. Discontinuation because the patient is unable to take the medication
   1. The reason for discontinuing was classified in this category when it was stated in the patients’ file that the patient was unable to take the medication.
4. Discontinuation because the patient was undoubtedly in the terminal stage, and it was clear that there was no longer an indication present.
   1. The reason for discontinuing was classified in this category when it was clear the patient was in a terminal stage. In such situation there was often opted for a comfort and care policy in which almost all medication was discontinued.
5. Discontinuation because of side-effects
   1. The reason for discontinuing was classified in this category when it was clearly stated in the patients’ file by their GP that the medication was discontinued because the patient experienced side-effects such as myalgia or gastro-intestinal complaints.
6. **Patient demographics**

Patient’s demographic included sex, age at time of death as described in paragraph 1.1. and existence of a life limiting illness as described in paragraph 1.2. Patients were divided into five categories: >55, 55-64, 65-74, 75-84, ≥85 years of age. Based on life-limiting illness, patients were subdivided into nine diagnosis categories as shown in appendix 1.

**Appendix 3 – ATC-codes**

The list below states all ATC-codes linked to the medication that was analysed in this study.

Fibrates, even though they are cholesterol-lowering, were not included in the analyses since they are not a first-line treatment in the Netherlands and therefore hardly ever prescribed.

List of Anatomical Therapeutic Chemical (ATC) classification codes^3^

| **ATC-code** | **Medication** |
| --- | --- |
| Cholesterol-lowering medications |  |
| C10AA01 | Simvastatin |
| C10AA03 | Pravastatin |
| C10AA04 | Fluvastatin |
| C10AA05 | Atorvastatin |
| C10AA07 | Rosuvastatin |
| C10AX09 | Ezetimibe |
| C10BA02 | Simvastatin and ezetimibe |
| Vitamins |  |
| A11AA03 | Multivitamins and other minerals, incl combinations |
| A11CC03 | Alfacalcidol |
| A11CC04 | Calcitriol |
| A11CC05 | Cholecalciferol |
| A11DA01 | Thiamine |
| A11E | Vitamin B-complex, incl combinations |
| A11EA | Vitamin B-complex, plain |
| A11EB | Vitamin B-complex, with vitamin C |
| A11GA01 | Ascorbic acid (Vit C) |
| A11HA02 | Pyridoxine (Vit B6) |
| A11JA | Combinations of vitamins |
| B03BA01 | Cyanocobalamin |
| B03BA03 | Hydroxocobalamin |
| B03BB01 | Folic acid |
| Calcium (A12A) |  |
| A12AA04 | Calcium Carbonate |
| A12AA20 | Calcium |
| A12AX | Calcium, combinations with vitamin D and/or other drugs |
| Bisphosphonates |  |
| M05BA02 | Clodrinic acid |
| M05BA04 | Alendronic acid |
| M05BA06 | Ibradronic acid |
| M05BA07 | Risedronic acid |
| M05BA08 | Zoledronic acid |
| M05BB03 | Alendronic acid and colecalciferol |
| M05BB05 | Alendronnic acid, Calcium and Colecalciferol, sequential |
| M05BX04 | Denosumab |

Literature

1. Nederlands Huisartsen Genootschap. ICPC [Internet]. Available from:<https://www.nhg.org/themas/artikelen/icpc>. Accessed 12^th^ of March 2021.
2. WHO. International Classification of Primary Care, 2nd edition ICPC-2 [Internet] Available from: <https://www.who.int/standards/classifications/other-classifications/international-classification-of-primary-care>. Accessed on 16th of March 2021.
3. WHO Collaboration Centre for Drug Statistics Methodology. ATC/DDD Index 2021 [Internet]. Available from:<https://www.whocc.no/atc_ddd_index/>. Accessed 1^st^ of March 2021.
